# Risk-factors for nodular hyperplasia of parathyroid glands in sHPT patients

**DOI:** 10.1371/journal.pone.0186093

**Published:** 2017-10-17

**Authors:** Mark D. Jäger, Michaela Serttas, Jan Beneke, Jörg A. Müller, Harald Schrem, Alexander Kaltenborn, Wolf Ramackers, Bastian P. Ringe, Jill Gwiasda, Wolfgang Tränkenschuh, Jürgen Klempnauer, Georg F. W. Scheumann

**Affiliations:** 1 Klinik für Allgemein-, Viszeral- und Minimal-Invasive Chirurgie, Städtisches Klinikum Wolfenbüttel gGmbH, Wolfenbüttel, Germany; 2 Klinik für Allgemein-, Viszeral- und Transplantationschirurgie, Medizinische Hochschule Hannover, Hannover, Germany; 3 Core Facility Quality Management in Transplantation, Integrated Research and Treatment Center Transplantation, Hannover Medical School, Hannover, Germany; 4 Klinik für Nuklearmedizin, Medizinische Hochschule Hannover, Hannover, Germany; 5 Klinik für Unfallchirurgie und Orthopädie, Bundeswehrkrankenhaus Westerstede, Westerstede, Germany; 6 Institut für Pathologie, Medizinische Hochschule Hannover, Hannover, Germany; Hospital Universitario de la Princesa, SPAIN

## Abstract

**Introduction:**

Nodular hyperplasia of parathyroid glands (PG) is the most probable cause of medical treatment failure in secondary hyperparathyroidism (sHPT). This prospective cohort study is located at the interface of medical and surgical consideration of sHPT treatment options and identifies risk-factors for nodular hyperplasia of PG.

**Material and methods:**

One-hundred-eight resected PG of 27 patients with a broad spectrum of sHPT severity were classified according to the degree of hyperplasia by histopathology. Twenty routinely gathered parameters from medical history, ultrasound findings of PG and laboratory results were analyzed for their influence on nodular hyperplasia of PG by risk-adjusted multivariable binary regression. A prognostic model for non-invasive assessment of PG was developed and used to weight the individual impact of identified risk-factors on the probability of nodular hyperplasia of single PG.

**Results:**

Independent risk-factors for nodular hyperplasia of single PG were duration of dialysis in years, PG volume in mm^3^ determined by ultrasound and serum level of parathyroid hormone in pg/mL. Multivariable analyses computed a model with an Area Under the Receiver Operative Curve of 0.857 (95%-CI:0.773–0.941) when predicting nodular hyperplasia of PG. Theoretical assessment of risk-factor interaction revealed that the duration of dialysis had the strongest influence on the probability of nodular hyperplasia of single PG.

**Conclusions:**

The three identified risk-factors (duration of dialysis, PG volume determined by ultrasound and serum level of parathyroid hormone) can be easily gathered in daily routine and could be used to non-invasively assess the probability of nodular hyperplasia of PG. This assessment would benefit from periodically collected data sets of PG changes during the course of sHPT, so that the choice of medical or surgical sHPT treatment could be adjusted more to the naturally changing type of histological PG lesion on an individually adopted basis in the future.

## Introduction

Parathyroid glands (PG) of patients suffering from secondary hyperparathyroidism (sHPT) due to chronic kidney disease are hyperplastic. The pattern of hyperplasia changes from a diffuse to a nodular one [1]. Nodular hyperplasia of PG exceeded the point of no return in pathological progression [2,3] and has been identified as the key issue for the failure of medical treatment in sHPT patients [4]. Even the application of calcimimetics, which is known to effectively reduce PTH secretion and PG volume in several patients [5–8], fails to control sHPT in patients with nodular hyperplasia of PG [9–11].

The non-invasive identification of nodular hyperplasia of PG could empower a critical consideration of treatment options like changing the drug treatment or progressing to interventional and surgical therapy [12–15]. Especially in the era of calcimimetics, it is imaginable that targeted treatment of severely diseased PG could be adequate when nodular hyperplasia can be linked to particular glands, potentially sparing patients from intolerably hypoparathyroidism after radical interventions. Therefore, a non-invasive assessment of individual PG might become more relevant for clinical follow-up and potentially individual decision making in sHPT patients [16].

So far, only few studies analyzed parameters associated with nodular hyperplasia of PG in a limited manner. Former large studies from Japan analyzing the relationship of PG size measured by ultrasound (US) and the histological pattern of PG confirmed by histology showed that a volume of PG larger than 300 mm^3^ respectively a diameter of PG longer than 8 mm predicted nodular hyperplasia [2,4,17]. These studies analyzed severely diseased sHPT patients with an average duration of dialysis about 13 years. Consequently these studies might not provide relevant data to assess changes in PG histopathology and associated pathophysiology during the course of sHPT as desirable in modern times using calcimimetics.

A study from Italy described that a high body mass index and female gender were associated with increased risk for nodular hyperplasia of PG in a Caucasian patient cohort [18]. Recently, more sophisticated US classifications were suggested to determine nodular hyperplasia of PG [19–21]. However, none of these studies analyzed both parameters from daily routine as well as data from detailed US of PG. Moreover, none of these studies combined the analysis of a broad spectrum of sHPT severity and a consequent histopathological evaluation of all detected PG. Thus, changes of PG during the course of sHPT and the influencing risk-factors could not be assessed so far. Last, but not least no study included a risk-adjusted stratification of the reported factors or any assessment of risk-factor interaction.

The aim of this prospective study was to identify risk-factors for nodular hyperplasia of PG in sHPT patients right at the interface of medical and surgical sHPT treatment–where modern interdisciplinary medicine should take place. For the first time, routinely available parameters of demographic and laboratory data as well as multiple variables from US of PG were brought together into multivariable analysis to identify independent risk-factors. Finally, a prognostic model was used to assess the interactive impact of the identified risk-factors on the probability of nodular hyperplasia of individual PG. The individual PG was consciously chosen as end-point, since the identification of treatment refractory PG might guide the decision for medical or surgical sHPT treatment options.

Thus, sHPT patients could probably be spared from continuous extensive drug application avoiding to prolong indefinitely a treatment with little chance of being successful and on the other hand from uncritical extensive surgical intervention causing intolerably hypoparathyroidism [22].

## Material and methods

### Setting and data collection

This prospective cohort study was performed at Hannover Medical School, Germany, a tertiary referral center for endocrine surgery between 2008 and 2010.

### Inclusion and exclusion criteria

Patients aged over 18 years with sHPT and chronic kidney failure which were scheduled for parathyroidectomy in our center were included. Patients who already had received a previous parathyreoidectomy were excluded.

### Patients

The study cohort contained 27 patients with chronic kidney diseases and sHPT, whereby a broad spectrum of sHPT severity was consciously covered.

Two of the patients did not need dialysis, but suffered from CKD stage 4 and mild sHPT. These patients received an early parathyroidectomy in the context of an indicated thyroidectomy when they did not yet receive any sHPT medication. Twenty-five patients dialyzed and were treated for sHPT using medical treatment, but no calcimimetics in eleven cases and using medical treatment including calcimimetics in fourteen patients.

### Ethics

The study was approved by the institutional ethics committee (Ethikkommission der Medizinischen Hochschule Hannover) at the 13. August 2007 and was conducted in accordance with the guidelines published in the Declaration of Helsinki. Informed consent was obtained from all patients.

### Variables and measurements

Laboratory values were gathered according to study protocol one day before surgery utilizing automated standard techniques in clinical routine practice.

**Fig 1:**
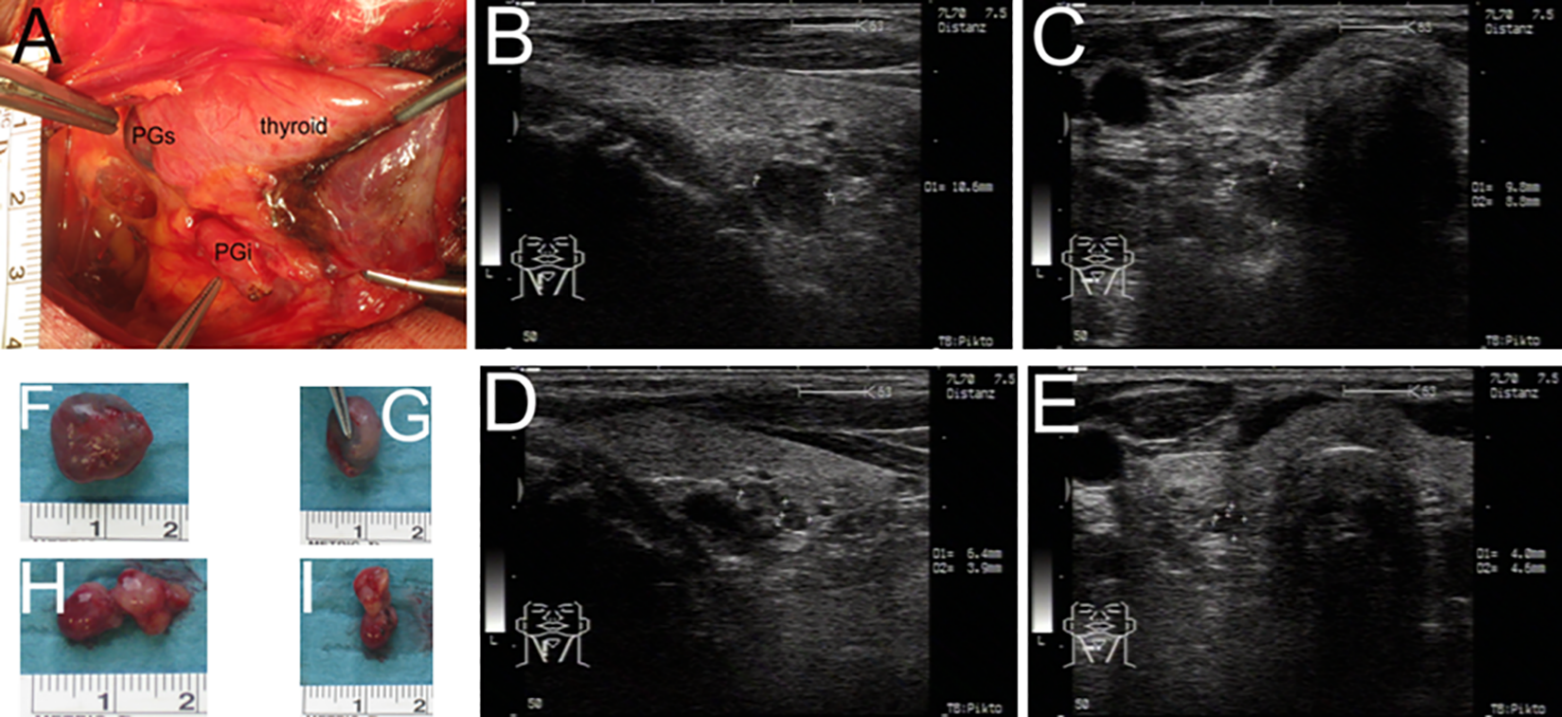
Topography and size of parathyroid glands at parathyroidectomy and ultrasound. Topography of the right-sided superior and inferior PG after luxation of the thyroid gland (A). Topography and measurement of corresponding PG by US: superior (B+C) and inferior (D+E). Volumes of PG were 479mm^3^ (PG superior) and 99mm^3^ (PG inferior) when calculated with US measurements. Documentation of PG size during surgery: superior (F+G) and inferior (H+I). Volumes of PG were 858mm^3^ (PG superior) and 293mm^3^ (PG inferior) when calculated with intra-operative measurements. PGs, superior parathyroid gland; PGi, inferior parathyroid gland.

Standardized US of the neck including Doppler imaging was performed by a single physician (JAM) with more than 20 years of experience in ultrasound of thyroid and parathyroid glands while being blinded to the statistical evaluation and results. US was performed one day before parathyreoidectomy using the Sonoline Sienna equipment (Siemens, Erlangen, Germany) with a linear array (7.5 MHz, 7 cm in length). Standardized photo-documentation included topographical anatomy of the thyroid gland, PG and surrounding structures. The detection of PG by US was matched with the topographical findings during parathyreoidectomy and confirmed by histology. Diameters of PG were measured in three dimensions, the largest of which was defined as length of PG in mm. The volume of PG was calculated by the formula for volume of an ellipsoid (a x b x c x π x 1/6) [23]. The echostructural pattern of PG was classified in an ordinal scale according to a former study published by Vulpio et al. (Vulpio classification: (1) hypoechoic homogeneous; (2) slightly heterogeneous; (3) highly heterogenous, and (4) nodular) [20]. Vulpio et al. assumed that stages 1 and 2 were associated with diffuse hyperplasia, whereas stages 3 and 4 mainly belong to nodular hyperplasia of PG. We applied this hypothesis by applying the binary variable echostructural pattern >2. Blood flow signals were classified in ordinal scale at periphery (P-Signal, 0 to 2) and in centre of PG (C-Signal, 0 to 2) according to former studies [19,20]. All parathyreoidectomies were performed by two experienced endocrine surgeons (MDJ, GFWS) simultaneously and included a cervical thymectomy to detect as most as possible PG. All located PG were documented exactly in correspondence to surrounding structures and the topographical anatomy was photo-documented (**Fig 1**). All PG were measured in three dimensions to calculate the volume [23]. Based on this detailed documentation, every lesion that was detected by US and the corresponding lesion documented during surgery could jointly be topographically correlated by four of the authors (MDJ, MS, JAM, GFWS).

The standard surgical procedure was a complete parathyroidectomy including cervical thymectomy with autografting of parathyroid tissue obtained from the 1 or 2 most morphologically conserved PG into the sternocleidomastoid muscle. This was performed in 24 patients where at least 4 PG were resected. In 3 patients only 3 PG could be detected despite cervical thymectomy so that autografting was not performed.

### Study end-point

Histologically confirmed nodular hyperplasia of parathyroid glands was defined as the primary study end-point.

### Histological classification of parathyroid glands

Every resected parathyroid gland was histologically examined at least at five different levels with the pathologist being fully unaware of the results of any other variable nor the prognostic model calculations. The pattern of parathyroid hyperplasia was classified as proposed by Tominaga et al. [2]. Based on this histological classification each PG was categorized as nodular or non-nodular hyperplasia as published before [17].

### Statistics

The required sample size for the current study was determined by a power analysis on the basis of the percentages of nodular hyperplastic PG found in previous studies (74%; 522 of 706 PG [4], 80%; 247 of 308 PG [17], 70%; 44 of 63 PG [19], 78%; 38 of 49 PG [24], and 93%; 52 of 56 PG [25]) using MedCalc version 16.7, Ostend, Belgium. Based on this information a sample size of more than 56 PG would lead to a power of at least 80%. This minimal sample size was increased to 70 PG with a complete dataset in purpose of reducing the beta-error.

Data is presented by mean values and standard deviations. Two-sided, unpaired Student’s *t*-tests and paired t-tests were used where appropriate. Correlations were calculated by the Pearson´s correlation coefficient. Univariable and multivariable binary regression analysis were used to determine risk-factors for nodular hyperplasia in PG. All statistically significant risk-factors (p<0.05) from univariable analysis were included in a multivariable risk-adjusted binary regression model, which was applied to identify independent risk-factors for nodular hyperplasia in PG.

To identify primary risk-factors for nodular hyperplasia, we first established a risk-index and investigated the significantly relevant parameters. Multivariable principal component analysis was applied to identify multi-collinearity between variables (correlation <0.500) for prognostic score design. A prognostic model was developed with the logit-link function in multivariable logistic regression accepting an alpha-level <0.200 in order to reduce the risk of overfitting. Results were assessed for model fit with Pearson, Deviance, and Hosmer-Lemeshow tests. The receiver operating characteristic (ROC) curve analysis was used to determine sensitivities and specificities of the developed prognostic model for the prediction of nodular hyperplasia [26]. Internal validation used backwards randomized bootstrapping as described before on randomized samples with 50–60% of the study population and subsequent ROC-curve analysis [27].

The individual impact of the identified risk-factors on the probability of nodular hyperplasia of PG was quantified using the prognostic model. To establish comparability, values of those factors were categorized and a standard unit for alteration was defined. Thereby, each risk-factor was separately altered by using 10 well defined steps within a range referring to the focus of the study as follows:

- duration of dialysis from 1 to 10 years by steps of 1 year,
- volume of PG measured by US from 100 to 1000 mm^3^ by steps of 100 mm^3^ and
- serum level of PTH from 1000 to 100 pg/mL by steps of 100 pg/mL.

While regarding each parameter separately, the others were set to a default reference level with duration of dialysis = 2 years, PG volume determined by US = 400 mm^3^, and PTH serum level = 600 pg/mL.

The computed risk equation of each altered variable was visualized into a graph.

Statistical analysis was performed using Minitab 16 (Minitab Inc., State College, Pennsylvania, USA) and SPSS 21.0 (IBM Corp., Armonk, NY, USA). P-values <0.050 were defined as significant.

## Results

### Patients and parathyroid glands

Demographic, clinical and laboratory values of the twenty-seven patients are summarized in Table 1.

**Table 1:** Demographic, clinical and laboratory characteristics of patients.

Female, n (%)	13 (48)
Age, y	55 (20–76,9)
Body mass index, kg/m^2^	26,5 (17,3–42,9)
Patients under dialysis, n (%)	25 (93)
Duration of dialysis, y	4,3 (0–15,8)
GFR, mL/min	< 15 (< 15–21)
Patients receiving calcimimetics, n (%)	14 (52)
PTH, pg/mL	525 (200–1613)
Calcium, mmol/L	2.41 (2,15–2,76)
Phosphorus, mmol/L	4,9 (2,4–9,9)
1,25-(OH)_2_-vitamin D, pmol/L	11,6 (3,1–56,5)

Data of the twenty-seven patients are shown as median (range) unless otherwise indicated.

GFR, glomerular filtration rate; PTH, parathyroid hormone

Overall 108 PG were resected at 27 parathyroidectomies (3 PG in 3 patients, 4 PG in 21 patients and 5 PG in another 3 patients). Pre-operative US detected 70 PG (detection rate 64.8%): 1 PG in 4 patients, 2 PG in 10 patients, 3 PG in 7 patients, 4 PG in 5 patients and finally 5 PG in 1 patient. Pre-operative US detected all resected PG in 6 patients. Nodular hyperplasia was present in 47 of 108 resected PG (43.5%) and in 34 of 70 PG pre-operatively detected by US (48.6%). Ten patients had no nodular PG and 4 patients had only nodular PG. Further characteristics of PG are given in Table 2. The volume of PG determined by US and during parathyreoidectomy were strongly correlated (r = 0.867), whereby volumes determined by US were significantly smaller as those measured during parathyreoidectomy (p<0.001, paired t-test).

**Table 2:** Characteristics of parathyroid glands.

	total	subgroup			
		1	2	3	*p*^a^
PG detected by US		yes	yes	no	
Kind of size measurement	at surgery	by US	at surgery	at surgery	
Number of PG, n	108	70	70	38	
Vol. of PG, mm^3^	838 ± 939	680 ± 851	1 033 ± 1 038	479 ± 579	0.003
PG nodular, n (%)	47 (43.5)	34 (48.6)	34 (48.6)	13 (34.2)	0.151^b^
Vol. of PG nodular, mm^3^	1 311 ± 1 171	1 061 ± 1 061	1 480 ± 1 250	868 ± 813	0.057
Vol. of PG non-nodular, mm^3^	474 ± 460	320 ± 305	612 ± 523	277 ± 248	0.004
*p* (vol. of PG nodular vs. non-nodular)	< 0.001	< 0.001	< 0.001	0.002	

Every PG detected at surgery was measured (column named total). Subgroups were defined by type of PG detection and by the kind of size measurement. Subgroups 1 and 2 contain only PG detected by pre-operative US and give data for measurement of PG volumes using pre-operative US or intra-operative 3-dimensional measurement. Subgroup 3 contains PG that were not pre-operatively detected by US, but measured intra-operatively after surgical detection.

Data are expressed as mean values ± standard deviation unless otherwise indicated.

PG, parathyroid gland; US, ultrasound; Vol, volume.

Significances were tested by two-sided, unpaired *t*-test.

^a^ Significance was tested between subgroup 2 and 3.

^b^ Significance was tested by two-sided Chi-square test.

*P* values of < 0.05 were considered statistically significant.

### Risk-factors for nodular hyperplasia of PG

Twenty variables were analyzed by univariable binary logistic regression analysis to identify possible associations with nodular hyperplasia of PG. Nine of those appeared to be significantly (p<0.05) and independently correlated with nodular hyperplasia (Table 3) and were included in subsequent multivariable analysis.

**Table 3:** Variables associated with nodular hyperplasia of individual parathyroid glands (univariable binary logistic regression analysis).

**Variables with significant influence**	***p***	**OR**	**95%-CI**
Age [years]	0.010	1.040	1.009–1.072
Duration of dialysis [years]	< 0.001	1.279	1.122–1.459
GFR [mL/min]	0.032	0.867	0.776–0.989
Phosphate [mmol/L]	0.011	0.366	0.170–0.791
PTH [pg/mL]	0.037	0.998	0.997–1.000
Length of PG in US [mm]	0.005	1.146	1.042–1.260
Volume of PG in US [mm^3^]	0.002	1.002	1.001–1.003
Volume of PG in US > 300mm^3^ [no = 0, yes = 1]	0.002	4.800	1.745–13.207
C-Signal [0–2]	0.013	3.463	1.303–9.207
**Variables without significant influence**	***p***	**OR**	**95%-CI**
Gender [male = 1, female = 2]	0.452	n.a.	n.a.
Body mass index [kg/m^2^]	0.809	n.a.	n.a.
Intake of cinacalcet [no = 0, yes = 1]	0.464	n.a.	n.a.
Intake of vitamin D or analogs [no =, yes = 1]	0.575	n.a.	n.a.
Calcium [mmol/L]	0.252	n.a.	n.a.
1,25-OH-vitamin D [pmol/L]	0.884	n.a.	n.a.
PG detected by US [no = 0, yes = 1]	0.151	n.a.	n.a.
Length of PG in US > 8mm [no = 0, yes =]	0.251	n.a.	n.a.
Echostructural pattern Vulpio classification [1–4]	0.421	n.a.	n.a.
Echostructural pattern > 2 [no = 0, yes = 1]	0.498	n.a.	n.a.
P-Signal [0–2]	0.748	n.a.	n.a.

Nodular hyperplasia in PG is the output variable (nodular = 1, non-nodular = 0). Binary and ordinal scale definitions are indicated for each variable.

OR, odds ratio; 95%-CI, 95% confidence interval; n.a., not announced; GFR, glomerular filtration rate; PTH, parathyroid hormone; PG, parathyroid gland; US, ultrasound; C-Signal, blood flow signal in the centre of PG; P-Signal, blood flow signal at the periphery of PG. *P* values of < 0.05 were considered statistically significant.

Remaining independent risk-factors for nodular hyperplasia in PG were the duration of dialysis in years, the volume of individual PG determined by US in mm^3^ and the serum level of PTH (Table 4).

**Table 4:** Independent risk factors for nodular hyperplasia of individual parathyroid glands (multivariable binary logistic regression analysis).

Variables	*p*	OR	95%-CI
Age [years]	0.907	n.a.	n.a.
Duration of dialysis [years]	0.015	1.394	1.067–1.821
GFR [mL/min]	0.815	n.a.	n.a.
Phosphate [mmol/L]	0.553	n.a.	n.a.
PTH [pg/mL]	0.049	0.995	0.989–1.000
Length of PG in US [mm]	0.915	n.a.	n.a.
Volume of PG in US [mm^3^]	0.010	1.003	1.001–1.005
Volume of PG in US > 300mm^3^ [no = 0, yes = 1]	0.976	n.a.	n.a.
C-Signal [0–2]	0.509	n.a.	n.a.

Nodular hyperplasia in individual PG is the output variable (nodular = 1, non-nodular = 0). Binary and ordinal scale definitions are indicated for each variable.

OR, odds ratio; 95%-CI, 95% confidence interval; n.a., not announced; GFR, glomerular filtration rate; PTH, parathyroid hormone; PG, parathyroid gland; US, ultrasound; C-Signal, blood flow signal in the centre of PG. *P* values of < 0.05 were considered statistically significant.

### Prognostic model predicting the probability of nodular hyperplasia of PG

The computed logit link function in multivariable binary regression used these three variables identified. Multicollinearity was precluded by preceding multivariable principal component analysis. Thus, the following prognostic model is proposed for the prediction of probability of nodular hyperplasia in percent:

[%]=Exp(Y)÷(1+Exp(Y))with

$$Y=-2,103+(0,002\times PG\;volume\;[{mm}^{3}])+(0,305\times duration\;of\;dialysis[years])+(-0,001\times PTH\;[pg/mL])$$

**Fig 2:**
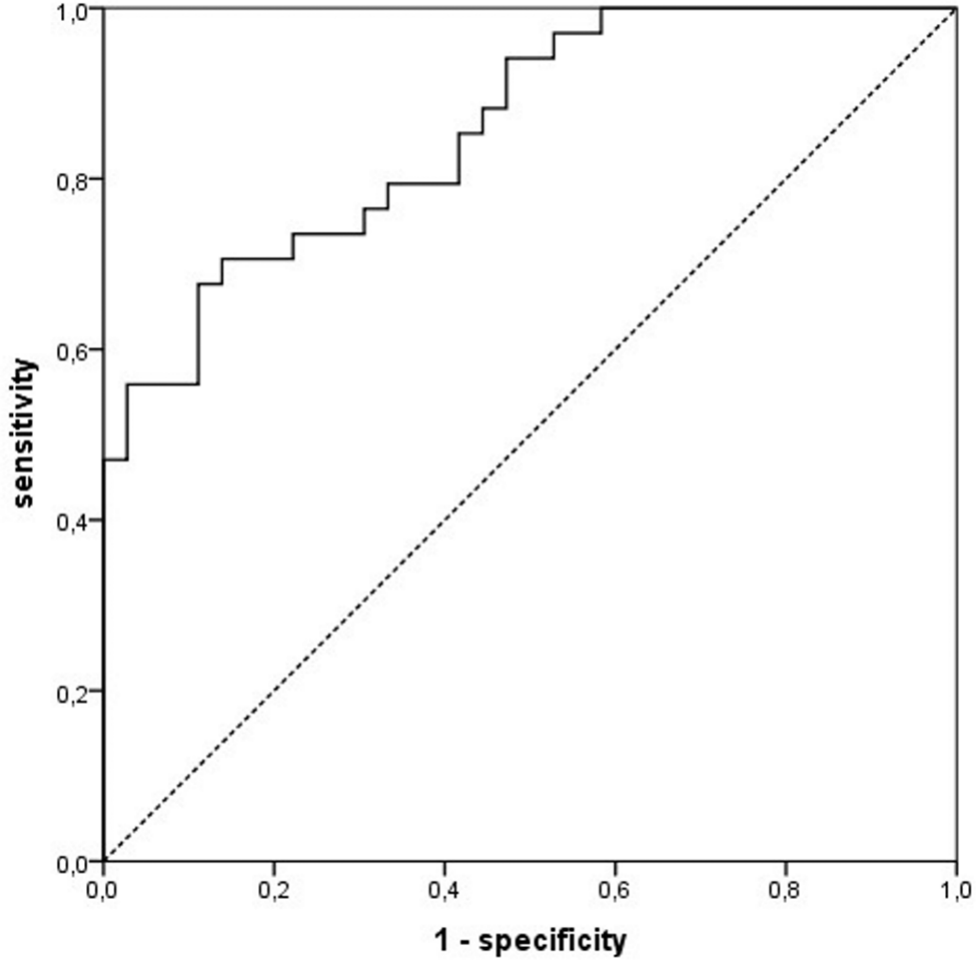
Receiver operating characteristic—curve for the prediction of nodular hyperplasia in individual parathyroid glands by the use of the prognostic model. The AUROC is 0.857 with a binominal exact 95% confidence interval: 0.773–0.941. The best Youden index determined a predicted probability of 52.9% as the cut-off value with best sensitivity and specificity for prediction of nodular hyperplasia in individual (specificity 86.1%, sensitivity 70.6%, overall correctness 78.3%). Good model fit was demonstrated by use of Pearson (0.75), Deviance (0.49), and Hosmer-Lemeshow tests (0.16).

The corresponding ROC-curve and the subsequent statistical evaluation of the reliability is demonstrated in **Fig 2**.

The flow of analyzed patients and PG through the study as well as the determination of the probability of nodular hyperplasia in individual PG as assessed with the proposed prognostic model is summarized in S1 Fig.

Internal validation was confirmed by randomized backwards bootstrapping which is summarized in the Table 5.

**Table 5:** Internal validation of the developed prognostic score using randomized bootstrapping.

Randomized % of cohort	AUROC	95%-CI
60	0.859	0.748–0.970
59	0.835	0.720–0.949
58	0.831	0.697–0.965
57	0.905	0.818–0.991
56	0.858	0.730–0.986
55	0.850	0.737–0.963
54	0.890	0.801–0.980
53	0.859	0.733–0.986
52	0.940	0.865–0.999
51	0.862	0.748–0.977
50	0.876	0.771–0.980

Shown are the results of internal validation of the developed prognostic model using randomized bootstrapping. It was validated eleven times with a random sample of 60–50% of the original study population and subsequent ROC-curve analysis. A successful validation was possible in every validation step.

A clinical example for the prediction of the probability of nodular hyperplasia in individual PG using the prognostic model is given in S2 Fig.

### Impact of individual risk-factors on the probability of nodular hyperplasia of PG

**Fig 3:**
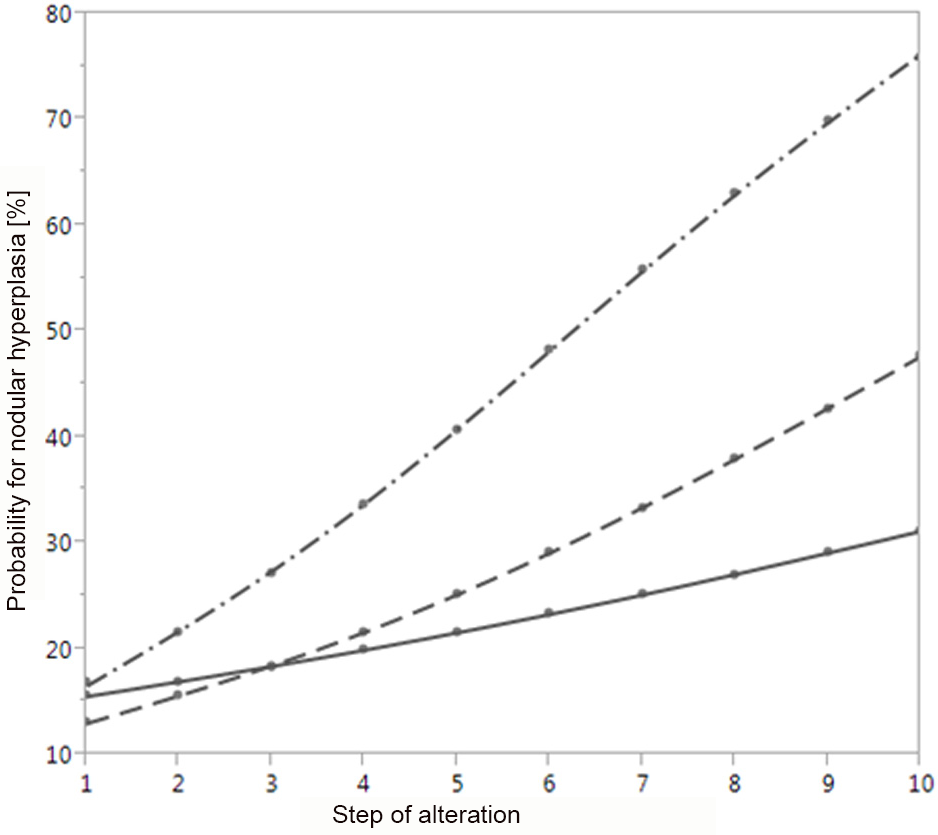
Probability of nodular hyperplasia of PG depending on values of individual risk-factors. Values of the duration of dialysis (−∙−∙−), PG volume measured by US (---) and serum levels of PTH (−−−) were categorized and standard units for a 10-step alteration were defined. The duration of dialysis was prolonged from 1 to 10 years by steps of 1 year. The PG volume determined by US was increased from 100 to 1.000 mm^3^ by steps of 100 mm^3^. The units of the 10-step alteration are given at the x-axis. The serum level of PTH was decreased from 1.000 to 100 pg/mL by steps of 100 pg/mL. While altering each parameter separately, the others were set to a default reference level with duration of dialysis = 2 years, PG volume determined by US = 400 mm^3^, and PTH serum level = 600 pg/mL. The calculated probability for nodular hyperplasia of PG is given in percent at the y-axis.

A visualization of the probabilities, which have been calculated by independently altered risk-factors using the prognostic model, is depicted in **Fig 3**. By categorizing and using a 10-step alteration of the individual factors it turned out that a stepwise prolongation of the duration of dialysis evoked the strongest increase in the probability of nodular hyperplasia of PG. A stepwise increase of PG volume resulted in a lesser slope of the probability whereas a stepwisely altered PTH serum level changed the probability only slightly. It has to be pointed out that by the structure of the computed risk equation the total probability is not directly additive.

## Discussion

The major strength of this study is the analysis of a broad spectrum of sHPT severity and the consequent histopathological classification of hyperplasia in all detected PG. The patient cohort covered the complete progression of sHPT ranging from patients with mild sHPT receiving no medical treatment to patients suffering from sHPT refractory to medical treatment including calcimimetics (Table 1).

We gained detailed data sets for every of the 108 PG detected by surgery. If a PG was not detected by pre-operative US, it was consequently excluded from regression analysis (S1 Fig). These were 13 nodular hyperplastic PG and 25 diffuse hyperplastic ones. Overall we could analyze a balanced proportion of nodular versus diffuse hyperplastic PG (34 versus 36) by regression analysis, what represents a unique composition of histologically confirmed PG hyperplasia compared to other studies [4,17,19,24,25]. These former studies analyzed mainly severely diseased sHPT patients or missed histology of PG from patients with mild sHPT. Thus, their proportion of nodular hyperplasia of PG was 70 to 93%.

Consequently, our study is unique by providing a balanced histopathological spectrum of PG as well as wide ranges of PG measurements (length, volume), of PTH levels and of the duration of dialysis. Thus, our unique setting elucidates for the first-time risk-factors for nodular hyperplasia of single PG right at the glance where its results should consequently be applied—at the interface of medical and surgical sHPT treatment to optimally adjust PTH levels within target ranges by interdisciplinary approaches as the circumstances require [22].

Our multivariable analyses identified the duration of dialysis, the serum level of PTH and the PG volume as determined by US as independent risk-factors for nodular hyperplasia of individual PG in sHPT patients. Thereby, our study considered all parameters previously suggested by univariable analyses elsewhere. Quite notably, this broad diversity of parameters was reduced to only one PG specific (PG volume) and two patient specific risk-factors (duration of dialysis, PTH level).

Previously described PG specific parameters in detail PG volume > 300 mm^3^, length of PG and C-signal assessed by US were confirmed by our univariable regression analyses, but failed as independent risk-factors when transferred to multivariable analyses [4,17,19]. More pretentious US parameters like categorization of echostructure or blood flow signaling failed as independent risk-factors in our study [19–21]. The simple parameter “volume of PG” as determined by US was the only PG specific risk-factor for nodular hyperplasia of single PG. Thus, an easy calculation of PG volume based on the three-dimensional measurement of PG by US is highly relevant for non-invasive assessment of PG pathology.

The anamnestic parameter “duration of dialysis” is a highly relevant independent risk-factor for nodular hyperplasia of single PG and is automatically known in every patient. Interestingly, laboratory variables, which are pathophysiologically involved in etiology and progression of PG hyperplasia like serum levels of calcium, phosphate and vitamin D, failed either in univariable or in multivariable regression analyses. In this study cohort, the variable “duration of dialysis” was superior to relevant laboratory variables. This might be caused by the fact, that dialysis therapy subsumes the multifarious long-term burden of CKD despite sHPT treatment and is less susceptible to quick volatile changes than laboratory values.

The serum level of PTH was also identified as independent risk-factor, but had only weak impact on the probability of nodular hyperplasia of PG. Thus, an alteration from 100 pg/mL to 1.000 pg/mL, which represents a dramatic shift from a value below the target range to a strongly pathological one, changed the probability of nodular hyperplasia of a singular PG only slightly as visualized in **Fig 3**. This result could be influenced by the diversified medical treatment of patients within our cohort that purposely compromised patients from CKD stage 4 without any sHPT treatment to dialysis patients with severe sHPT refractory to calcimimetic treatment.

A new scope for clinical routine was added to our first-time analysis of independent risk-factors by considering interactions and dependencies between our identified three risk-factors. Therefore, default reference levels for each risk-factor were purposely chosen below any limits for surgical treatment to give a realistic impression of the probability of nodular hyperplasia of PG in patients, which are usually not transferred to surgery. Assuming separate alteration of individual risk-factors in this theorized patients with relatively mild sHPT, results show that the probability of nodular hyperplasia in PG is most severely increased by the “duration of dialysis”. Thus, prolonged duration of dialysis fundamentally alters the perspective upon PG volume determined by US and serum level of PTH. This high impact on the probability of nodular hyperplasia of PG by the duration of dialysis is statistically proven for the first time and therefore newly based on evidence. In clinical routine it should be considered that the longer patients undergo dialysis treatment, the more carefully PG volume should be assessed by US especially when sHPT medication is escalated and non-invasive assessment of PG pathology is desired (16). Thereby, standard US equipment as used by our study is sufficient to assess single PG in sHPT patients. Since the reliable identification of PG by US might be even more difficult than measuring the PG size in three dimensions, it seems recommendable to become familiar with routinely US investigations of PG.

Our study has some limitations. Although our sample size meets the power calculations as indicated in the methods section and the scientific context, it is likely that a larger cohort and an external validation of the prognostic model would enable a higher reliability. The US device utilized in this study was not on the highest, but on a standard technological level supplying significantly valuable data, which might qualify the current findings as applicable in common clinical routine [16,28]. The binary end-point defined by histologically confirmed nodular hyperplasia of singular PG fulfills clearly the requirement for a statistical analysis of independent risk-factors, but is only a well correlating surrogate marker for drug refractiveness of PG in sHPT patients [4,9–11].

Perspectively, the evaluation of the probability of nodular hyperplasia in individual PG would be most reasonable in patients being refractory to calcimimetic treatment [29]. The respect for hypoparathyroidism and contraindications for extensive surgery are examples encouraging the consideration of local ablation or targeted surgery of most likely nodular hyperplastic PG in such patients [15,23,30]. Although higher probability for nodular hyperplasia would always be assumed and calculated for larger PG within the same patient, prolonged dialysis treatment also puts smaller PG at a relevant risk for nodular hyperplasia.

In conclusion, non-invasive assessment of PG pathology could be easily performed in sHPT patients using the identified independent risk-factors. Thus, usage of the PG volume determined by US measurement, the duration of dialysis and the serum level of PTH might be sufficient to estimate the probability of nodular hyperplasia in PG. The suggested non-invasive assessment would strongly benefit from periodically collected data sets of PG changes during the course of sHPT. Thus, the choice of medical or surgical sHPT treatment could be adjusted more to the naturally changing type of histological PG lesion on an individually adopted basis in order to avoid a prolonged indefinite pharmacological treatment with little chance of being successful or an uncritical surgical overtreatment causing intolerably hypoparathyroidism. Modern sHPT treatment could even combine surgery of all PG with highly probable nodular hyperplasia and subsequently the pharmacological control of almost normal and remaining PG in some cases. Such a procedure could be imaginable in approximately a quarter of our analyzed cases, which possessed at least one parathyroid gland with a volume of less than 100 mm^3^ and without nodular hyperplasia, but would still require a complete intra-operative detection and confirmation in size of all PG as well as a reliable collaboration of experienced endocrine surgeons and nephrologists. The appropriateness and long-term results of such an interdisciplinary combined approach would need to be proven in another prospective study.

## Supporting information

S1 Fig Flow chart of patients and parathyroid glands through study and model development. Thirty-eight of 108 PG were not detected by ultrasound (US) and were excluded from model development. The cut-off probability to predict nodular hyperplasia of PG was 52.9% as determined by the Youden index with the best sensitivity (70.6%) and specificity (86.1%).
(PDF)

S2 Fig Documentation of preoperative ultrasound results of the thyroid and parathyroid glands in patient #14 and the predicted probability of nodular hyperplasia in individual PG including the histopathological assessment. This sketch of US examination belongs to a 55 year old male who dialyzed for 1.6 years. He had a PTH serum level of 620 pg/mL despite sHPT treatment with phosphate binders and vitamin D analogs (subgroup 2). The pre-operative examination by US detected four PG (= NSD) in this patient–one in each quadrant at the thyroid gland. The three dimensional measurements of the PG (= NSD) were given in centimeter and were documented by hand-writing in the sketch. The calculation of PG volumes used these measurement and resulted in 3770 mm^3^ (upper right PG: 2,4 x 2 x 1,5 cm), 3780 mm^3^ (lower right PG: 2 x 1,9 x 1,9 cm), 346 mm^3^ (upper left PG: 1,1 x 1 x 0,6 cm) and 2375 mm^3^ (lower left PG: 1,2 x 1,8 x 2,1 cm).
The calculated probabilities for nodular hyperplasia using the prognostic model were: 99.5% (upper right PG), 99.5% (lower right PG), 17.6% (upper left PG) and 92.5% (lower left PG).
The post-operative histopathological assessment of individual PG is given as N for nodular hyperplasia and as D for diffuse hyperplasia.
(GIF)
