# Supplementary material for: Risk-factors for nodular hyperplasia of parathyroid glands in sHPT patients
Source: PLoS One. 2017 Oct 17;12(10):e0186093. doi: 10.1371/journal.pone.0186093 (PMC5645091; doi:10.1371/journal.pone.0186093)
Supplement: S1 Fig — Thirty-eight of 108 PG were not detected by ultrasound (US) and were excluded from model development. The cut-off probability to predict nodular hyperplasia of PG was 52.9% as determined by the Youden index with the best sensitivity (70.6%) and specificity (86.1%). (PDF) [file pone.0186093.s001.pdf]

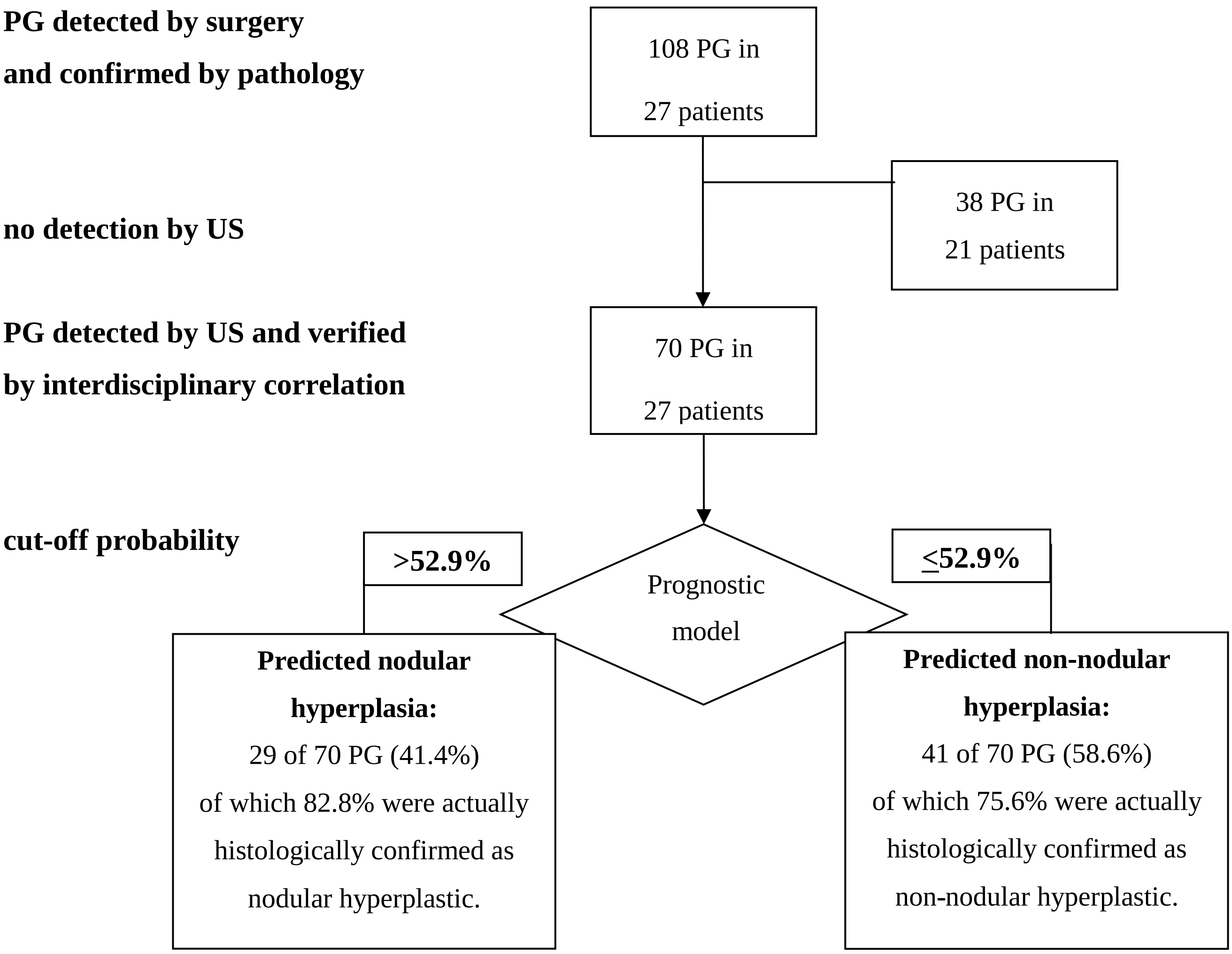
PG detected by surgery
and confirmed by pathology
108 PG in
27 patients
no detection by US
38 PG in
21 patients
PG detected by US and verified
by interdisciplinary correlation
70 PG in
27 patients
cut-off probability
>52.9%
Prognostic
model
≤52.9%
Predicted nodular
hyperplasia:
29 of 70 PG (41.4%)
of which 82.8% were actually
histologically confirmed as
nodular hyperplastic.
Predicted non-nodular
hyperplasia:
41 of 70 PG (58.6%)
of which 75.6% were actually
histologically confirmed as
non-nodular hyperplastic.
