# Supplementary figures and images for: Risk-factors for nodular hyperplasia of parathyroid glands in sHPT patients
Source: PLoS One. 2017 Oct 17;12(10):e0186093. doi: 10.1371/journal.pone.0186093 (PMC5645091; doi:10.1371/journal.pone.0186093)

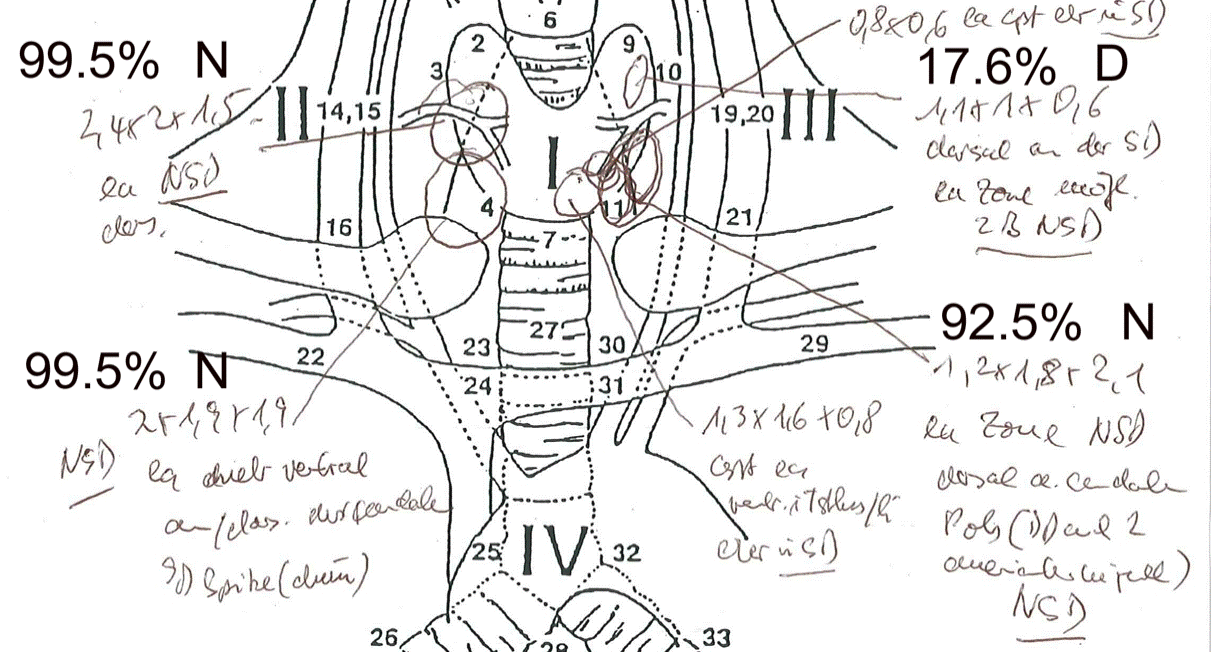

Supplement: S2 Fig — This sketch of US examination belongs to a 55 year old male who dialyzed for 1.6 years. He had a PTH serum level of 620 pg/mL despite sHPT treatment with phosphate binders and vitamin D analogs (subgroup 2). The pre-operative examination by US detected four PG (= NSD) in this patient–one in each quadrant at the thyroid gland. The three dimensional measurements of the PG (= NSD) were given in centimeter and were documented by hand-writing in the sketch. The calculation of PG volumes used these measurement and resulted in 3770 mm3 (upper right PG: 2,4 x 2 x 1,5 cm), 3780 mm3 (lower right PG: 2 x 1,9 x 1,9 cm), 346 mm3 (upper left PG: 1,1 x 1 x 0,6 cm) and 2375 mm3 (lower left PG: 1,2 x 1,8 x 2,1 cm). The calculated probabilities for nodular hyperplasia using the prognostic model were: 99.5% (upper right PG), 99.5% (lower right PG), 17.6% (upper left PG) and 92.5% (lower left PG). The post-operative histopathological assessment of individual PG is given as N for nodular hyperplasia and as D for diffuse hyperplasia. (GIF) [file pone.0186093.s002.gif]
